# Migration background, eating disorder symptoms and healthcare service utilisation: findings from the Stockholm Public Health Cohort

**DOI:** 10.1192/bjo.2023.599

**Published:** 2023-11-03

**Authors:** Mattias Strand, Sofie Bäärnhielm, Peeter Fredlund, Boel Brynedal, Elisabeth Welch

**Affiliations:** Centre for Psychiatry Research, Department of Clinical Neuroscience, Karolinska Institutet, Sweden; and Transcultural Centre, Northern Stockholm Psychiatry, Stockholm Health Care Services, Region Stockholm, Sweden; Centre for Epidemiology and Community Medicine, Region Stockholm, Sweden; and Department of Global Public Health, Karolinska Institutet, Sweden; Centre for Psychiatry Research, Department of Clinical Neuroscience, Karolinska Institutet, Sweden; and Department of Women's and Children's Health, Uppsala University, Sweden

**Keywords:** Anorexia nervosa, bulimia nervosa, feeding or eating disorders, transcultural psychiatry, stigma and discrimination

## Abstract

**Background:**

From a global perspective, eating disorders are increasingly common, probably because of societal transformation and improved detection. However, research on the impact of migration on the development of eating disorders is scarce, and previously reported results are conflicting.

**Aims:**

To explore if eating disorder symptom prevalence varies according to birth region, parents’ birth region and neighbourhood characteristics, and analyse if the observed patterns match the likelihood of being in specialist treatment.

**Method:**

This study uses data from a large population-based health survey (*N* = 47 662) among adults in Stockholm, Sweden. A general linear model for complex samples, including adjustment for gender and age, was used to explore self-reported eating disorder symptoms. Odds ratios were calculated for individual symptoms.

**Results:**

Eating disorder symptoms are substantially more common in individuals born abroad, especially for migrants from a non-European country. This holds true for all surveyed symptoms, including restrictive eating (odds ratio 5.5, 95% CI 4.5–6.7), compensatory vomiting (odds ratio 6.1, 95% CI 4.6–8.0), loss-of-control eating (odds ratio 2.6, 95% CI 2.3–3.1) and preoccupation with food (odds ratio 2.3, 95% CI 1.9–2.8). Likewise, symptoms are more common in individuals with both parents born abroad and individuals living in districts with a high percentage of migrant residents. A gap exists between district-level symptom scores and the likelihood of being in specialist eating disorder treatment.

**Conclusions:**

These findings call for oversight of current outreach strategies, and highlight the need for efforts to reduce stigma and increase eating disorder symptom recognition in broader groups.

Eating disorders such as anorexia nervosa, bulimia nervosa and binge eating disorder used to be seen as conditions that mostly affect White women from socioeconomically privileged families – during the 1980s, eating disorders were even described as a uniquely Western ‘culture-bound syndrome’.^1^ Today, it is well-established that this stereotypical view is erroneous and outdated: eating disorders can affect people from all social strata, regardless of gender, ethnicity or socioeconomic background,^2^ and give rise to a substantial global burden of disease.^3^ From a global perspective, eating disorders are becoming more common,^4^ not least in East and South-East Asia,^5^ which is probably because of both societal transformation and improved detection by healthcare providers.

Unfortunately, there is scarce research on the impact of migration on the development of eating disorders and on treatment-seeking. An Australian study reported a significantly lower eating disorder prevalence in first-generation immigrants compared with the Australian-born population.^6^ Likewise, a study utilising Swedish and Danish registered diagnoses found a lower prevalence of all eating disorders among individuals with a migration background;^7^ however, if service utilisation differs between groups, these findings may simply reflect underdetection in healthcare. In contrast, a Spanish population-based study showed that female immigrant adolescents were more likely to display eating disorder symptoms compared with Spanish-born adolescents.^8^ Notably, the only published study that reported an increased likelihood of treatment-seeking for eating disorders in migrants is a second study based on Australian data.^9^

In contrast, there is a substantial body of research on eating disorders in people of Black and minority ethnicity. A number of studies, mostly from the USA and UK, have shown that the prevalence of eating disorders is just as high or sometimes even higher among people of Black and minority ethnicity compared with the White majority population.^10–12^ The exact diagnostic patterns may vary, however; for example, eating disorders characterised by loss of control and overeating, such as bulimia nervosa and binge eating disorder, appear to be more common among Black and Latinx populations in the USA and among South Asians in the UK, compared with the White majority population, whereas this is generally not the case for anorexia nervosa.^11,13^ Despite these findings, fewer people of Black and minority ethnicity still receive specialist eating disorder treatment;^14,15^ this has been called a ‘blind spot’ in the field.^16^ Depending on migration context and history, these findings may or may not be relevant for migrant populations.^17^ In the USA, with its long history of forced and voluntary immigration, many people of Black and minority ethnicity do not have any recent migration background. To a certain degree, this holds true for countries such as the UK and France as well, although here large groups of migrants from former colonies have arrived after 1945. In Sweden, where the present study was conducted, migration from non-European countries is a much more recent phenomenon, and although the groups are not completely interchangeable, there is a substantial overlap between people of Black and minority ethnicity and persons with a non-European migration background (as defined below).

A number of barriers affect access to mental healthcare among migrant groups.^18,19^ For example, so-called health literacy – a term describing a basic knowledge about, and ability to act upon, medical symptoms – may be limited,^20^ in particular among an older generation that grew up in a cultural context where mental health was not discussed openly.^21^ This, in turn, may give rise to feelings of shame and stigma, further constraining help-seeking.^21,22^ Structural issues associated with legal and socioeconomic status, such as worries about out-of-pocket healthcare expenditures, loss of work income, transport and childcare, may also be crucial barriers to accessing healthcare in general^18,19^ as well as specialist eating disorder treatment.^22^ A small number of studies, mostly from the USA, have explored specific barriers to specialist eating disorder treatment for people of Black and minority ethnicity, many of which may also be relevant for persons with a migration background. For example, a prevailing stereotypical view of eating disorders as solely affecting White women may limit symptom recognition and help-seeking. Black patients can describe how they, when partaking in eating disorder treatment, also have to battle an internalised image of eating disorders as something that ‘should not’ affect them as a group.^22^ These stereotypical views also affect healthcare providers. Several studies show that people of Black and minority ethnicity that seek help for eating disorder symptoms are less often referred for specialist treatment by their general practitioners compared with White people with an identical clinical picture.^23,24^ Similar findings are reported from vignette studies where fictional patient cases in which only patient ethnicity varies have been distributed to medical doctors and psychologists: case descriptions involving a Black or minority ethnic person are more seldom identified as an eating disorder and do not result in an offer of adequate treatment to the same extent as case descriptions involving White patients.^25,26^ Legitimate fear of being rejected by healthcare providers may thus also affect help-seeking patterns.^22,24^

## Objective

This study uses data from a large population-based public health survey in Stockholm, Sweden, to explore self-reported eating disorder symptoms among adults with a migration background compared with the Swedish-born population. The objectives of the study are (a) to explore if the prevalence of eating disorder symptoms varies according to birth region, parents’ birth region and neighbourhood characteristics; and (b) to analyse if the observed district-level patterns match the likelihood of being in specialist eating disorder treatment. For comparison, measures on mental distress and suicidality in the various groups are also analysed. Based on previous research, we hypothesise that we will observe a substantial burden of eating disorder symptoms in individuals with a migration background on a level similar to what is seen in the Swedish-born population. We also hypothesise that this burden will not be mirrored by actual treatment-seeking patterns, especially among individuals with a migration background.

## Method

### Study population

The Stockholm Public Health Cohort (SPHC) is a population-based longitudinal cohort study based on the Stockholm Regional Council public health survey ‘Hälsa Stockholm’, distributed every fourth year since 1990, with the purpose of informing on determinants and consequences of the current burden of disease in the population.^27^ Participants are randomly selected individuals from the adult population of Stockholm County (total population of 2.4 million, of which 36% have a migration background as defined below). Data in the SPHC are collected by postal questionnaires (with optional responding online) covering a large number of health-related variables. Questionnaires are available in Swedish, English, Arabic and Polish. Self-reported data have been complemented by information from Swedish national longitudinal health and sociodemographic data registries. To date, the SPHC comprises roughly 140 000 participants. In the present study, the 2014 wave of the SPHC (*N* = 50 157 respondents) was utilised for cross-sectional analyses; this wave was chosen because survey items about eating disorder symptoms have not been included in subsequent waves. Only respondents with a known district of residence within the Stockholm County were included in the data-set. Since this particular cohort included only those adults (≥18 years) that had been surveyed in the previous wave 4 years earlier, respondent age was ≥22 years.

In the SPHC, calibration weights have been used to correct for the stratified random sample design, as well as for systematic non-response at baseline and follow-up. Statistics Sweden calculated these calibration weights based on available auxiliary variables from national registries, and their association with selected survey variables and the probability of participating in the survey. Auxiliary variables included gender, age, country of birth, educational level, disability allowance and area of residence (i.e. sampling strata). A detailed description of the weighting procedure and the considerations behind it is available from Statistics Sweden.^28^

An *a priori* power analysis using G*Power software version 3.1. for Windows (Heinrich Heine University Düsseldorf, Germany; see https://www.psychologie.hhu.de/arbeitsgruppen/allgemeine-psychologie-und-arbeitspsychologie/gpower) indicated that to achieve 95% power for detecting a small effect size (*f*^2^ = 0.02) at a significance level of *α* = 0.05 for a fixed-model linear multiple regression including three predictors, the required sample size was *N* = 863. Thus, the obtained sample size of *N* = 47 662 was adequate to test the study hypotheses.

### Exposures

The three main exposures used in the analysis of the survey data were region of birth, parents’ region of birth and percentage of residents with a migration background in the neighbourhood. Regarding region of birth, respondents were categorised as either born in Sweden, Europe (other than Sweden) or a non-European country. A further subgroup analysis included the world regions described in Supplementary Table 1 available at https://doi.org/10.1192/bjo.2023.599.

Regarding parents’ region of birth, respondents were categorised as having two Swedish-born parents, one parent born abroad or two parents born abroad. A further subgroup analysis included only those respondents with two parents born in a non-European country.

Regarding neighbourhood characteristics, respondents were categorised as living in a neighbourhood in which residents with a migration background comprised <20%, 20–40% or >40% of the population. Since there is no established standard for reporting percentage of residents with a migration background in the neighbourhood, this model with three quintile categories was chosen so as to reflect the actual population composition in the municipalities and districts in the Stockholm County in a reasonable way.

Data on age, gender, country/region of birth and parents’ region of birth were extracted through record linkage with the Multi-Generational Register at Statistics Sweden. It can be noted that for parents’ region of birth, there was initially a relatively large amount of data missing, at 23%. After consultation with Statistics Sweden and to increase accuracy, an assumption was made that those born after 1950 whose parents’ region of birth was missing had parents that were born outside of Sweden, because of the fact that missing data on Swedish-born parents in the registers is in the range of 0–2% for this age group, compared with up to 86% for foreign-born parents. This reduced the missing data to 9.9%. For calculations on the subgroup with parents born outside of Europe, this assumption could not be made.

The Stockholm County is divided into 26 municipalities. One of these municipalities, the City of Stockholm (the most populous municipality in Sweden), is further divided into 14 districts. Thus, a total of 39 geographic strata (hereafter referred to as districts) were used in this study. Data for the year 2014 on district characteristics in terms of the percentage of residents with a migration background were obtained from Statistics Sweden^29^ and the City of Stockholm statistical yearbook.^30^ In both databases, people with a migration background were defined as those either born abroad or having two foreign-born parents; it can be noted that this is a relatively strict definition of migration background, since the term may also be used to describe those with only one foreign-born parent or one or more foreign-born grandparents.

### Outcomes

Regarding eating disorder symptoms, parts of the SCOFF scale were employed. The SCOFF questionnaire^31^ is composed of five questions on disordered eating, three of which (‘Do you make yourself sick because you feel uncomfortably full?’, ‘Do you worry that you have lost control over how much you eat?’ and ‘Would you say that food dominates your life?’; hereafter referred to as SCOFF3) are included in the 2014 wave of the SPHC. SCOFF3 is scored dichotomously, where a ‘yes’ answer gives a score of 1 for that item and a ‘no’ answer a score of 0, for a possible sum score of 0–3. The SCOFF scale has demonstrated good validity as a screening tool for clinically relevant eating disorders.^31^ Furthermore, the 2014 wave of the SPHC includes one question on restrictive eating (‘On a scale from 1 to 8, where 1 means no dietary restraint (I eat whatever I want, whenever I want) and 8 means total dietary restraint (I always limit food intake and never give in), how would you rate yourself?’). For the purpose of this study, a conservative measure was used by which only answers 7 or 8 were coded as ‘yes’. This item was then added to the SCOFF3 score for a combined eating disorder score, ranging from 0 to 4. Body mass index (BMI) was calculated with the respondents’ answers for weight and height.

Although the study objectives concern eating disorder symptoms, measures of more general mental distress and suicide were also included to determine whether the observed patterns were exclusive for eating disorder morbidity or part of an overall greater vulnerability for psychopathology. Levels of mental distress were measured with the 12-item General Health Questionnaire (GHQ-12), which is one of the most widely used general measures of psychiatric well-being and a common screening tool for mental disorders. The GHQ-12 has been shown to be valid and reliable for use in the Stockholm population.^32^ Each item in the GHQ-12 has four Likert-scale response alternatives; for this study, a customary bimodal scoring model was used (by which the four response alternatives are scored 0-0-1-1), for a possible sum score of 0–12. In addition, the survey includes two questions on suicidality (‘Have you ever seriously considered taking your life, and perhaps even planned how to go about it?’ and ‘Have you ever made a suicide attempt?’). These two items were combined into a single bimodal suicidality score whereby a positive answer on any of them were scored as 1.

Data on service utilisation was obtained from the Stockholm Regional Council internal healthcare data analysis system, accessed through the analysis tool QlikView version 12 for Windows (QilkTech International, King of Prussia, PA, USA; see https://www.qlik.com/us/products/qlikview). Here, district-level data on the number of patients and number of out-patient visits to the government-run specialist eating disorder service Stockholm Centre from Eating Disorders (SCÄ) in the relevant age span for the year 2014 were extracted. Data on district-level eating disorder diagnoses were also extracted. SCÄ is a public healthcare provider serving the entire Stockholm County, with only minor patient fees in consonance with all Swedish public healthcare. According to the national quality register for eating disorder treatment, SCÄ served 73% of all patients seeking specialist eating disorder treatment in Stockholm County in 2014.^33^ For comparison, district-level data for the year 2014 on the number of patients and number of out-patient visits to one of the five public psychiatric hospital clinics in Stockholm for depressive or anxiety disorders were extracted; these disorders were assumed to best reflect an elevated GHQ-12 score. To examine any changes in patterns over time, corresponding service utilisation data for the year 2021 were also extracted. Importantly, it should be noted that the QlikView data represent actual patients, and that there has not been any record linkage between these data and the SPHC. Therefore, no conclusions can be drawn regarding the treatment-seeking behaviours of individual survey respondents; available data merely allow us to compare district-level variation in symptoms with the likelihood of treatment-seeking in the various districts.

### Statistical analysis

For SCOFF3, the combined eating disorder score, the GHQ-12 score, the suicidality score and BMI, weighted estimated means for the various exposures as well as mean differences were calculated, along with 95% confidence intervals, using a general linear model for complex samples. In these analyses, the Swedish-born group, the group with two parents born in Sweden or the group from a neighbourhood with <20% of residents with a migration background were used as reference groups, respectively. Because of well-known gender- and age-related differences in eating disorder morbidity, gender and age were included *a priori* as confounders in a further regression analysis model; furthermore, the non-adjusted findings are presented for the full sample as well as for women and men separately. Similarly, for district-level data on the likelihood of receiving treatment, eating disorder symptom scores and registered eating disorder diagnosis for those in treatment, estimated means as well as mean differences were calculated, along with 95% confidence intervals. In this analysis, the group from a neighbourhood with <20% of residents with a migration background was used as reference group. Relationships between neighbourhood characteristics and district-level registered eating disorder diagnosis for those in treatment, between district-level eating disorder symptom scores and treatment-seeking, and between a district's geographical distance (in kilometres) to the specialist eating disorder service and treatment-seeking were also explored with a general linear model. For the individual SCOFF items and the restrictive eating item, weighted crosstab analyses were performed for the various population groups. For these crosstab calculations, odds ratios with 95% confidence intervals were calculated, in which the same groups as above were used as reference groups. Further subgroup analyses were performed for birth region categorised according to the world regions described in Supplementary Table 1 and for those with two parents born in a non-European country. Software SAS version 9.4 for Windows was used for data extraction from the SPHC and associated registers. Software IBM SPSS Statistics version 28 for Windows and the SPSS Complex Samples module were used for all statistical analyses.

### Preregistration and ethics

The study protocol has been preregistered on the Open Science Framework (osf.io/acfdy). It may be noted that a statistical approach utilising pairwise independent samples *t*-tests for differences in means was initially planned for comparisons across groups; however, this approach was abandoned in favour of the regression model described in more detail above. The authors assert that all procedures contributing to this work comply with the ethical standards of the relevant national and institutional committees on human experimentation and with the Helsinki Declaration of 1975, as revised in 2008. All procedures involving human participants were approved by the Swedish Ethical Review Authority (approval number 2021-05935-01). All participants in the SPHC have given their informed consent to the preservation of their national registration number, future contacts and record linkages. In reporting the findings of the study, we have adhered to the Strengthening the Reporting of Observational Studies in Epidemiology (STROBE) statement on improving the quality of reporting of observational studies.^34^

## Results

Descriptive data on survey respondent characteristics, along with weighted estimated, are shown in Table 1; a further description of the sample by levels of exposure is provided in Supplementary Table 2. Tables 2–4 show weighted data on disordered eating, mental distress and BMI in relation to birth region, parents’ birth region and neighbourhood population characteristics, respectively. The corresponding models adjusted for gender and age are shown in Supplementary Tables 3–5; as seen, these *a priori* included confounders do indeed affect eating disorder symptom levels. Data on individual eating disorder symptom items for the various groups are shown in Table 5. Subgroup analyses for birth region and for those with two parents born in a non-European country are shown in Supplementary Tables 6 and 7, respectively.

**Table 1:** Description of the survey data

	*N*	%	Weighted %
Total number of respondents	47 662		
Gender			
Female	27 134	56.9	50.8
Male	20 528	43.1	49.2
Age groups			
22–29 years	1456	3.1	15.7
30–44 years	8623	18.1	30.3
45–66 years	20 936	43.9	35.3
>67 years	16 647	34.9	18.7
BMI groups			
<18.5 kg/m^2^	625	1.3	1.6
18.5–24.9 kg/m^2^	23 161	48.6	52.8
25.0–29.9 kg/m^2^	16 048	33.7	33.8
≥30.0 kg/m^2^	5503	11.5	11.8
Missing data	2325	4.9	Not applicable
Region of birth			
Sweden	40 855	85.7	73.6
Europe (other than Sweden)	4664	9.8	12.7
Non-European countries	2098	4.4	13.7
Missing data	45	0.1	Not applicable
Parent background			
Both parents born in Sweden	31 161	65.4	60.9
One parent born abroad	4719	9.9	10.4
Both parents born abroad	7061	14.8	28.7
Missing data	4721	9.9	Not applicable
Neighbourhood population			
<20% with migration background	20 235	42.5	33.9
20–40% with migration background	23 321	48.9	53.0
>40% with migration background	4106	8.6	13.1

BMI, body mass index.

**Table 2:** Region of birth in relation to disordered eating, mental distress and body mass index

	Sweden	Europe (other than Sweden)			Non-European countries		
	Mean (95% CI)	Mean (95% CI)	Mean difference^a^ (95% CI)	*P-*value	Mean (95% CI)	Mean difference^a^ (95% CI)	*P-*value
Total population							
SCOFF3 score	0.15 (0.14–0.15)	0.20 (0.18–0.22)	0.05 (0.03–0.07)	<0.001	0.37 (0.33–0.41)	0.22 (0.18–0.26)	<0.001
Combined eating disorder score	0.17 (0.16–0.18)	0.23 (0.21–0.25)	0.06 (0.04–0.09)	<0.001	0.48 (0.44–0.53)	0.31 (0.27–0.36)	<0.001
GHQ-12 score	1.67 (1.62–1.73)	1.52 (1.40–1.64)	−0.15 (−0.28 to −0.02)	0.021	1.96 (1.80–2.11)	0.28 (0.12–0.45)	<0.001
Suicidality score	0.15 (0.14–0.16)	0.13 (0.12–0.15)	−0.02 (−0.03 to 0.00)	0.044	0.15 (0.13–0.17)	0.00 (−0.02 to 0.02)	0.855
BMI	25.0 (24.9–25.0)	25.9 (25.7–26.0)	0.91 (0.73–1.08)	<0.001	25.7 (25.5–26.0)	0.76 (0.51–1.00)	<0.001
Women							
SCOFF3 score	0.20 (0.19–0.21)	0.22 (0.19–0.25)	0.02 (−0.01 to 0.05)	0.243	0.43 (0.37–0.48)	0.23 (0.17–0.29)	<0.001
Combined eating disorder score	0.22 (0.21–0.24)	0.25 (0.22–0.28)	0.03 (0.00–0.06)	0.080	0.54 (0.47–0.60)	0.31 (0.24–0.38)	<0.001
GHQ-12 score	1.88 (1.82–1.95)	1.71 (1.55–1.86)	−0.18 (−0.35 to −0.01)	0.041	2.35 (2.12–2.58)	0.47 (0.23–0.71)	<0.001
Suicidality score	0.16 (0.16–0.17)	0.14 (0.12–0.16)	−0.02 (−0.04 to 0.00)	0.021	0.20 (0.16–0.23)	0.03 (0.00–0.06)	0.050
BMI	24.3 (24.2–24.4)	25.4 (25.2–25.6)	1.08 (0.86–1.31)	<0.001	25.0 (24.7–25.3)	0.67 (0.36–0.98)	<0.001
Men							
SCOFF3 score	0.09 (0.09–0.10)	0.17 (0.14–0.20)	0.07 (0.04–0.10)	<0.001	0.31 (0.25–0.36)	0.21 (0.16–0.27)	<0.001
Combined eating disorder score	0.11 (0.10–0.12)	0.20 (0.17–0.23)	0.09 (0.06–0.12)	<0.001	0.42 (0.36–0.48)	0.31 (0.25–0.37)	<0.001
GHQ-12 score	1.47 (1.38–1.55)	1.27 (1.09–1.45)	−0.20 (−0.40 to 0.00)	0.052	1.53 (1.32–1.73)	0.06 (−0.16 to 0.28)	0.594
Suicidality score	0.14 (0.12–0.15)	0.12 (0.10–0.15)	−0.01 (−0.04 to 0.01)	0.341	0.10 (0.08–0.13)	−0.03 (−0.06 to −0.01)	0.019
BMI	25.6 (25.5–25.7)	26.5 (26.3–26.7)	0.91 (0.64–1.17)	<0.001	26.5 (26.1–26.8)	0.89 (0.51–1.26)	<0.001

GHQ-12, 12-item General Health Questionnaire; BMI, body mass index.

a. Compared with the Swedish-born group as reference.

**Table 3:** Parent background in relation to disordered eating, mental distress and body mass index

	Both parents born in Sweden	One parent born abroad			Both parents born abroad		
	Mean (95% CI)	Mean (95% CI)	Mean difference^a^ (95% CI)	*P-*value	Mean (95% CI)	Mean difference^a^ (95% CI)	*P-*value
Total population							
SCOFF3 score	0.14 (0.13–0.15)	0.18 (0.15–0.20)	0.04 (0.01–0.07)	0.005	0.30 (0.27–0.32)	0.16 (0.14–0.18)	<0.001
Combined eating disorder score	0.16 (0.15–0.17)	0.20 (0.17–0.23)	0.04 (0.01–0.07)	0.010	0.37 (0.34–0.40)	0.21 (0.18–0.24)	<0.001
GHQ-12 score	1.64 (1.58–1.70)	1.93 (1.75–2.10)	0.28 (0.10–0.47)	0.002	1.78 (1.68–1.88)	0.14 (0.02–0.26)	0.018
Suicidality score	0.15 (0.14–0.16)	0.17 (0.16–0.19)	0.03 (0.01–0.05)	0.013	0.16 (0.14–0.17)	0.01 (−0.01 to 0.02)	0.344
BMI	25.0 (24.9–25.0)	24.9 (24.7–25–1)	−0.06 (−0.29 to 0.05)	0.609	25.8 (25.6–26.0)	0.82 (0.64–1.01)	<0.001
Women							
SCOFF3 score	0.19 (0.18–0.21)	0.25 (0.21–0.29)	0.06 (0.01–0.10)	0.015	0.34 (0.31–0.37)	0.15 (0.11–0.18)	<0.001
Combined eating disorder score	0.22 (0.20–0.23)	0.27 (0.23–0.32)	0.05 (0.01–0.10)	0.026	0.41 (0.37–0.45)	0.19 (0.15–0.23)	<0.001
GHQ-12 score	1.84 (1.77–1.91)	2.23 (1.99–2.46)	0.39 (0.15–0.63)	0.002	2.08 (1.94–2.21)	0.24 (0.08–0.39)	0.002
Suicidality score	0.16 (0.15–0.17)	0.21 (0.18–0.24)	0.05 (0.02–0.08)	0.002	0.18 (0.16–0.20)	0.02 (0.00–0.04)	0.055
BMI	24.3 (24.2–24.4)	24.3 (24.0–24.6)	−0.07 (−0.39 to 0.24)	0.643	25.1 (24.9–25.3)	0.74 (0.53–0.94)	<0.001
Men							
SCOFF3 score	0.09 (0.08–0.10)	0.10 (0.07–0.13)	0.02 (−0.01 to 0.05)	0.263	0.25 (0.21–0.28)	0.16 (0.13–0.19)	<0.001
Combined eating disorder score	0.10 (0.09–0.11)	0.12 (0.09–0.15)	0.02 (−0.02 to 0.05)	0.271	0.32 (0.29–0.36)	0.22 (0.18–0.26)	<0.001
GHQ-12 score	1.46 (1.36–1.56)	1.61 (1.36–1.86)	0.16 (−0.11 to 0.42)	0.260	1.44 (1.30–1.58)	−0.02 (−0.19 to 0.15)	0.838
Suicidality score	0.14 (0.12–0.15)	0.14 (0.11–0.17)	0.01 (−0.03 to 0.03)	0.755	0.13 (0.11–0.14)	−0.01 (−0.03 to 0.01)	0.371
BMI	25.6 (25.4–25.7)	25.6 (25.2–25.9)	0.02 (−0.33 to 0.37)	0.912	26.6 (26.3–26.9)	1.05 (0.74–1.36)	<0.001

GHQ-12, 12-item General Health Questionnaire; BMI, body mass index.

a. Compared with the group with two parents born in Sweden as reference.

**Table 4:** Neighbourhood population in relation to disordered eating, mental distress and body mass index

	<20% with migration background	20–40% with migration background			>40% with migration background		
	Mean (95% CI)	Mean (95% CI)	Mean difference^a^ (95% CI)	*P-*value	Mean (95% CI)	Mean difference^a^ (95% CI)	*P-*value
Total population							
SCOFF3 score	0.14 (0.13–0.16)	0.18 (0.17–0.19)	0.04 (0.02–0.05)	<0.001	0.28 (0.25–0.31)	0.14 (0.10–0.17)	<0.001
Combined eating disorder score	0.17 (0.16–0.18)	0.22 (0.20–0.23)	0.05 (0.03–0.06)	<0.001	0.34 (0.31–0.38)	0.18 (0.14–0.21)	<0.001
GHQ-12 score	1.61 (1.54–1.68)	1.71 (1.64–1.78)	0.10 (0.00–0.20)	0.048	1.78 (1.63–1.92)	0.17 (0.00–0.33)	0.048
Suicidality score	0.14 (0.14–0.15)	0.15 (0.14–0.16)	0.00 (−0.01 to 0.02)	0.640	0.15 (0.13–0.17)	0.01 (−0.01 to 0.03)	0.420
BMI	24.9 (24.8–25.0)	25.1 (25.0–25.2)	0.20 (0.06–0.33)	0.004	26.2 (26.0–26.4)	1.26 (1.02–1.51)	<0.001
Women							
SCOFF3 score	0.19 (0.17–0.21)	0.23 (0.22–0.25)	0.04 (0.02–0.07)	0.002	0.33 (0.28–0.37)	0.13 (0.08–0.18)	<0.001
Combined eating disorder score	0.22 (0.20–0.25)	0.27 (0.25–0.29)	0.05 (0.02–0.08)	<0.001	0.39 (0.34–0.44)	0.16 (0.11–0.22)	<0.001
GHQ-12 score	1.83 (1.74–1.93)	1.92 (1.84–2.01)	0.09 (−0.03 to 0.22)	0.148	2.21 (1.91–2.33)	0.29 (0.06–0.52)	0.012
Suicidality score	0.16 (0.15–0.17)	0.16 (0.15–0.18)	0.00 (−0.01 to 0.02)	0.606	0.17 (0.15–0.20)	0.01 (−0.02 to 0.04)	0.396
BMI	24.3 (24.2–24.4)	24.5 (24.4–24.6)	0.20 (0.04–0.37)	0.015	25.7 (25.4–26.1)	1.47 (1.14–1.79)	<0.001
Men							
SCOFF3 score	0.09 (0.08–0.10)	0.13 (0.11–0.14)	0.04 (0.02–0.05)	<0.001	0.23 (0.19–0.28)	0.14 (0.09–0.19)	<0.001
Combined eating disorder score	0.11 (0.10–0.12)	0.16 (0.14–0.17)	0.05 (0.03–0.07)	<0.001	0.30 (0.25–0.35)	0.19 (0.14–0.24)	<0.001
GHQ-12 score	1.38 (1.27–1.49)	1.50 (1.39–1.60)	0.11 (−0.04 to 0.26)	0.143	1.43 (1.23–1.64)	0.05 (−0.18 to 0.28)	0.656
Suicidality score	0.13 (0.11–0.14)	0.13 (0.12–0.14)	0.00 (−0.02 to 0.02)	0.815	0.13 (0.11–0.16)	0.01 (−0.03 to 0.04)	0.717
BMI	25.6 (25.5–25.7)	25.7 (25.6–25.9)	0.16 (−0.05 to 0.37)	0.143	26.6 (26.3–26.9)	1.03 (0.67–1.39)	<0.001

GHQ-12, 12-item General Health Questionnaire; BMI, body mass index.

a. Compared with the group with <20% population with migration background as reference.

**Table 5:** SCOFF items and restrictive eating in the various groups

	Born in Sweden	Born in Europe (other than Sweden)		Born in non-European countries	
	% ‘Yes’ (95% CI)	% ‘Yes’ (95% CI)	Odds ratio^a^ (95% CI)	% ‘Yes’ (95% CI)	Odds ratio^a^ (95% CI)
SCOFF item					
Compensatory vomiting	1.1 (0.9–1.3)	2.6 (1.9–3.5)	2.5 (1.8–3.5)	6.1 (5.0–7.5)	6.1 (4.6–8.0)
Loss-of-control eating	7.9 (7.4–8.4)	9.6 (8.5–10.9)	1.2 (1.1–1.4)	18.4 (16.4–20.6)	2.6 (2.3–3.1)
Preoccupation with food	5.7 (5.3–6.2)	7.5 (6.5–8.5)	1.3 (1.1–1.6)	12.3 (10.6–14.3)	2.3 (1.9–2.8)
Restrictive eating	2.2 (2.0–2.5)	4.0 (3.3–4.8)	1.8 (1.4–2.3)	11.1 (9.5–12.9)	5.5 (4.5–6.7)
	Both parents born in Sweden	One parent born abroad		Both parents born abroad	
	% ‘Yes’ (95% CI)	% ‘Yes’ (95% CI)	Odds ratio^a^ (95% CI)	% ‘Yes’ (95% CI)	Odds ratio^a^ (95% CI)
SCOFF item					
Compensatory vomiting	0.9 (0.7–1.1.)	1.4 (0.8–2.5)	1.6 (0.9–2.9)	4.4 (3.8–5.2)	5.2 (4.0–6.8)
Loss-of-control eating	7.6 (7.0–8.2)	9.8 (8.3–11.5)	1.3 (1.1–1.6)	15.0 (13.8–16.3)	2.2 (1.9–2.4)
Preoccupation with food	5.4 (4.9–5.8)	6.5 (5.3–8.0)	1.2 (1.0–1.6)	10.3 (9.3–11.5)	2.0 (1.8–2.4)
Restrictive eating	2.0 (1.8–2.3)	2.0 (1.4–2.8)	1.0 (0.6–1.4)	7.2 (6.4–8.2)	3.8 (3.1–4.6)
	Area with <20% with migration background	Area with 20–40% with migration background		Area with >40% with migration background	
	% ‘Yes’ (95% CI)	% ‘Yes’ (95% CI)	Odds ratio^a^ (95% CI)	% ‘Yes’ (95% CI)	Odds ratio^a^ (95% CI)
SCOFF item					
Compensatory vomiting	1.1 (0.9–1.4)	1.9 (1.6–2.2)	1.7 (1.3–2.3)	4.2 (3.2–5.5)	4.0 (2.8–5.7)
Loss-of-control eating	7.6 (6.9–8.4)	9.6 (9.0–10.3)	1.3 (1.1–1.5)	13.9 (12.2–15.8)	2.0 (1.6–2.4)
Preoccupation with food	5.7 (5.1–6.3)	6.7 (6.2–7.3)	1.2 (1.0–1.4)	9.9 (8.5–11.6)	1.8 (1.5–2.2)
Restrictive eating	2.6 (2.2–3.0)	3.6 (3.2–4.0)	1.4 (1.2–1.7)	6.4 (5.1–7.8)	2.6 (2.0–3.4)

a. Compared with the Swedish-born group, the group with two parents born in Sweden or the group with <20% population with migration background as references, respectively.

In sum, eating disorder symptoms among adults in Stockholm County are more prevalent in individuals born outside of Sweden, especially so in those born outside of Europe. Likewise, eating disorder symptoms are more prevalent in individuals for whom both parents were born abroad. Moreover, there is an increase in eating disorder symptom prevalence as the district percentage of residents with a migration background increases. Adjusting for gender and age strengthens these differences slightly. The patterns for the GHQ-12 scores are more varied. For suicidality, few differences are found. The above findings are present for both women and men, but women display a larger magnitude of eating disorder symptom scores throughout.

Regarding the subgroup analyses, those born in Nordic countries other than Sweden display similar eating disorder scores as Swedish-born individuals; however, their mean GHQ-12 scores are lower. In contrast, eating disorder scores are elevated for those born in Africa, North America and South America compared with Swedish-born individuals. In particular, individuals born in Asia display markedly elevated eating disorder scores. Likewise, the patterns seen in Table 5 are more pronounced for individuals born in Asia. Notably, individuals born in Africa display the highest prevalence of restrictive eating. Regarding parent background, the patterns seen in Tables 3 and 5 are even more pronounced for individuals with two parents born in a non-European country.

District-level patterns of treatment-seeking are shown in Table 6 and illustrated in Supplementary Fig. 1. In sum, individuals in neighbourhoods with 20–40% of residents with a migration background tend to receive slightly more specialist eating disorder treatment compared with those in neighbourhoods with <20% of residents with a migration background, whereas those in neighbourhoods with >40% of residents with a migration background receive substantially less; all of these differences are non-significant by any standard. There is also a tendency for individuals in neighbourhoods with 20–40% of residents with a migration background to receive substantially more general psychiatric treatment than the other groups. There is an overall tendency for neighbourhoods with high eating disorder symptom prevalence to have fewer patients in specialist eating disorder treatment (*B* = −160.9; 95% CI −327.0 to 5.1; *P* = 0.057). Scatterplot analysis reveals that the four districts whose residents are most likely to receive specialist eating disorder treatment all have relatively low mean combined eating disorder scores; these districts also have relatively few residents with a migration background. On the contrary, the five districts with the highest mean combined eating disorder scores are all among the districts in which residents are least likely to receive treatment; these districts also have a high or very high percentage of residents with a migration background. Individuals from districts with longer geographical distance to the specialist eating disorder service are more seldom in treatment (*B* = −2.0; 95% CI −2.6 to −1.3; *P* = <0.001); however, this distance is not related to the percentage of the population with a migration background in a certain district (*B* = −11.6; 95% CI −40.3 to 17.0; *P* = 0.415), indicating that geographical distance is not more of an obstacle for individuals with a migration background. Patients from neighbourhoods with a high percentage of residents with a migration background are no more likely to receive a bulimia nervosa or binge eating disorder diagnosis as compared with an anorexia nervosa diagnosis (*B* = 0.02; 95% CI −0.20 to 0.25; *P* = 0.834).

**Table 6:** Neighbourhood population in relation to number of patients and visits in specialist treatment

	<20% with migration background	20–40% with migration background			>40% with migration background		
	Mean (95% CI)	Mean (95% CI)	Mean difference^a^ (95% CI)	*P-*value	Mean (95% CI)	Mean difference^a^ (95% CI)	*P-*value
Specialist eating disorder treatment							
Number of patients^b^	81.7 (62.1–101.2)	87.7 (71.3–104.1)	6.0 (−19.5 to 31.6)	0.651	56.3 (23.5–89.1)	−25.4 (−63.5 to 12.8)	0.194
Number of visits^b^	873.3 (594.6–1152.0)	1052.8 (819.7–1286.0)	179.6 (−183.8 to 542.9)	0.343	562.7 (96.4–1029.0)	−310.6 (−853.8 to 232.7)	0.252
General psychiatric specialist treatment							
Number of patients^b^	486.8 (231.6–741.9)	865.0 (651.5–1078.4)	378.2 (45.6–710.9)	0.032	559.4 (132.5–986.3)	72.7 (−424.7 to 570.0)	0.779
Number of visits^b^	2297.1 (766.0–3828.3)	5137.3 (3856.2–6418.4)	2840.1 (843.7–4836.5)	0.010	2398.7 (−163.4 to 4960.9)	101.6 (−2883.2 to 3086.4)	0.933

a. Compared with the group with <20% population with migration background as reference.

b. Per 100 000 inhabitants.

The district-level demographic composition in terms of migration background has not changed substantially since year 2014. Likewise, an analysis of data on specialist eating disorder treatment for the year 2021 shows that there were no major changes in the overall district-level patterns, i.e. on average, residents in districts in which a large percentage of the population have a migration background are no more or less likely to receive specialist eating disorder treatment in year 2021 compared with year 2014.

## Discussion

This study shows that, over and above what was hypothesised, eating disorder symptoms among adults in Stockholm, Sweden, are substantially more common in individuals born abroad, especially so for those who have migrated from a non-European country. Notably, the highest eating disorder symptom scores are found among those born in Asia. Likewise, eating disorder symptoms are substantially more common in individuals for whom both parents were born abroad and in individuals living in a district with a high percentage of residents with a migration background. As comparison, levels of mental distress in a more general sense do not differ for most groups, although there is an overall trend toward higher scores among individuals with a migration background. Very few differences between groups are found in terms of suicidality.

Importantly, the likelihood of district residents receiving specialist eating disorder treatment is not related to the district-level occurrence of eating disorder symptoms. Notably, residents of the five districts with the highest eating disorder symptom burden – all of which are also districts where a large share of the population has a migration background – are some of the least likely to receive treatment. Thus, our findings establish the existence of a substantial treatment gap, as hypothesised.

Previous Swedish research has shown that mental disorders such as schizophrenia and other psychotic disorders are more common among individuals with a migration background, regardless of refugee status.^18,35^ Post-traumatic stress disorder^36,37^ and autism^38^ are also more common among migrants in Sweden, whereas substance use^37^ and suicide^36,39^ tend to be less common and mirror the patterns in the individual's country of origin, at least during the first decade in Sweden. Considering the increased prevalence of several mental disorders, it may not be very surprising that eating disorder symptoms are also more common in individuals with a migration background, despite the fact that eating disorders have typically been conceptualised as ‘Western’ or ‘White’ disorders. For example, the association between traumatic experiences and the development of eating disorders – and bulimia nervosa and binge eating disorder in particular – is well established and may account for some of the observed risk.^40,41^ Furthermore, socioeconomic disadvantage, stress and discrimination, which are also more common among individuals with a migration background, may increase the risk of disordered eating behaviours, including those associated with obesity.^42,43^ Hypothetically, experiences of having a physical appearance that differs from that of the White majority population may give rise to body image concerns. Such concerns have been reported in migrant groups, although the existing literature on body dissatisfaction in racial and ethnic minorities is ambiguous.^44^

As described earlier, there is some evidence that eating disorders characterised by loss of control and overeating, such as bulimia nervosa and binge eating disorder, are more common among people of Black and minority ethnicity, whereas anorexia nervosa is less common than in the White population. In our findings, there were no clear district-level patterns in terms of eating disorder diagnosis in those patients that did partake in specialist eating disorder treatment. However, if the observed higher levels of eating disorder symptoms among individuals with a migration background reflect a ‘hidden’ burden of disease, it is reasonable to assume that it may heavily involve cases of bulimia nervosa and binge eating disorder, since these eating disorders typically go unrecognised for a longer period of time.^45^ Our finding that mean BMI was higher among those born abroad and in districts with many residents with a migration background supports this interpretation, and is mirrored by international research.^46^ There is recent evidence that so-called food insecurity (i.e. an enduring limited access to affordable and nutritious foods, which is more common in underprivileged groups) is associated with disordered eating, especially loss of control and binge eating.^43^ This may, for example, result from an oscillating ‘feast-or-famine’ tendency, in which food intake varies heavily as availability fluctuates (e.g. peaking after receiving a pay check), which can contribute to a vicious cycle of restriction and overeating characteristic of bulimia nervosa and binge eating disorder. Perhaps somewhat surprisingly for a society that is often perceived as relatively egalitarian, food insecurity has been shown to be fairly common in Sweden – in fact, even more so than in countries such as the USA, UK and France (although data are not always readily comparable).^47^

Swedish research has also shown that despite their increased burden of mental illness, individuals with a migration background tend to utilise less psychiatric care during their first decade in the country; over time, however, their service utilisation increases, so that migrants with a longer duration of stay use more psychiatric care than the Swedish-born population.^48^ Notably, migrants and children of migrants in Sweden are more likely to be compulsorily admitted at first admission for psychotic disorders,^49^ possibly implying that they have not been able to access mental healthcare at an earlier stage of illness. There may be several potential barriers to healthcare for migrants, some of which we outlined previously regarding how they relate to eating disorders: health illiteracy, shame and stigma, fear of being rejected by healthcare providers, limited economic resources, a lack of reliable interpretation services, etc. Swedish data indicate that levels of health literacy, trust and individual agency tend to be lower among individuals with a migration background, and that this group may have to rely more on assistance in navigating the healthcare system.^50^ Since eating disorder symptoms, not least those associated with loss of control and overeating, are often perceived as highly stigmatising,^21,22,45^ many may be reluctant to ask for such assistance. This reluctance may be even more pronounced in a migrant context, in part because of an unfortunate tendency of ‘blaming’ ill health on individual shortcomings among underprivileged groups in much public health campaigning.^51^

Another possible explanation is that individuals with a migration background may more often be treated for their eating disorder symptoms in a primary care setting, and that they therefore more seldom require or seek specialist treatment. However, Swedish studies indicate that people with a migration background tend to experience a lower satisfaction with primary care, and that they have more limited continuous access to a primary care physician.^52^ Moreover, research from the USA and the UK shows that primary care physicians more often fail to identify disordered eating in patients with a minority background.^23,24^ In Sweden, migrant children and adolescents with an eating disorder are no more likely to receive their diagnosis in primary care than in specialist care compared with non-migrant children.^53^ Thus, it seems highly unlikely that the availability of thorough eating disorder treatment at a primary care level is better for this group than for the rest of the population.

Although the calibration weights take a number of socioeconomic covariates into account, we have not specifically controlled our findings for potential socioeconomic confounders such as household income or education level; an individual's socioeconomic status in Sweden may be a consequence of their migrant status, and hence on the causal pathway between exposure and outcome. Also, it is important to keep in mind that the objective of this study was not to explore causality behind disordered eating, but to find out if the prevalence of eating disorder symptoms varies according to region of birth and migration background. We can conclude that, contrary to stereotypical views of eating disorders, disordered eating is more common in individuals with a migration background compared with the Swedish-born population in Stockholm; whether this is primarily attributable to socioeconomic disadvantages, experiences of exile and trauma, racism and discrimination, or any other reason is beyond the scope of this study.

### Strengths and limitations

This study adds to the relatively limited research literature examining disordered eating among migrants. The SPHC is one of the largest available public health cohorts of its kind, allowing for statistically robust analysis of health-related behaviours. The survey data in the SPHC has been complemented by information from Swedish longitudinal health and sociodemographic data registries, which are generally considered as being of very high quality in terms of scope and reliability.^27^ Data from the SPHC have been used in large number of epidemiological studies on various health-related topics. Moreover, unique access to district-level service utilisation data from the Stockholm Regional Council internal healthcare data analysis system allows us to compare symptom levels with treatment-seeking patterns.

Even so, the findings reported here should be interpreted in light of a number of limitations. Despite a sophisticated use of calibration weights in the SPHC to account for differences in non-response rates etc., there may still be some type of selection bias unaccounted for that leads to inflated eating disorder scores in respondents with a migration background. In general, however, individuals with poor mental health tend to be less motivated to participate in survey interviews,^54^ and it is unclear why this pattern would be different among those with a migration background specifically. Regarding respondent language, the survey was available in English, Arabic and Polish translations; however, very few chose to use the Arabic or Polish versions,^28^ and it can therefore be concluded that a large majority of respondents had a functional understanding of either Swedish or English. This, as well as the fact that there was no alternative for illiterate individuals, may have skewed the results in ways that cannot be fully compensated for by the weighting process.

The studies from the USA and the UK have usually analysed differences in eating disorder morbidity across various racial or ethnic groups.^10–15^ In Sweden, however, national regulations generally prohibit the registration of race and ethnicity, even for research purposes. Therefore, in this study we have grouped respondents according to their region of birth and the demographic characteristics of their district of residence. We use the term migration background, which is more common in a European context, but has also been criticised on the grounds of being too vague and/or as simply operating as a euphemism for racial categories.^55^ Of course, being born outside of Europe, for instance, does not imply that one is necessarily Black or minority ethnic. However, for reference, the five largest non-European migrant groups in Sweden in 2014 were Iraqi, Iranian, Syrian, Somali and Thai,^29^ and it is reasonable to assume that a major share of respondents with a migration background in this study are also people of Black and minority ethnicity.

The present study includes only respondents ≥22 years of age, whereas eating disorders often have an earlier age of onset. However, this is certainly not always the case. Both bulimia nervosa and binge eating disorder tend to occur at a slightly higher age,^4^ and there are indications that eating disorders are generally overlooked among middle- and older-aged individuals. As shown above, although the overall patterns regarding disordered eating were the same in all age groups, there was an age gradient by which the magnitude of eating disorder symptoms were greater for younger age groups. It is unlikely that the inclusion of even younger respondents would have led to markedly different results.

Naturally, the subjective presence of disordered eating behaviours does not mean that respondents necessarily fulfil diagnostic criteria for an eating disorder, although the SCOFF scale is commonly used as a screening tool in the assessment of eating disorders. Thus, we can only draw conclusions about self-reported symptom levels in the population. However, as seen in Table 5, scores on all four items that make up the combined eating disorder score were elevated among respondents with a migration background, suggesting that there is no specific symptom item (or misinterpretation of any specific item) that can explain the differences.

The internal healthcare data analysis system used to assess treatment-seeking patterns only include data for SCÄ, operated by the Stockholm Regional Council. SCÄ is one of the largest specialist eating disorder services in the world; however, there are also a small number of additional specialist eating disorder services in the region, run by a private healthcare provider under contract with the Stockholm Regional Council. Hypothetically, the patterns of service utilisation for the private-run services could differ from those of the government-run services. However, none of these services are situated in a district characterised by a high burden of eating disorder symptoms or by a high percentage of residents with a migration background as detailed by our data, which makes it unlikely that they attract a larger share of patients with a migration background.

The latest available data on eating disorder symptoms in the SPHC is from the 2014 survey. In the context of a study like this, data from 8 years ago can hardly be considered ancient; however, it is of course possible that the symptom levels in the various population groups analysed here have changed since data collection. However, for the measures that we were able to follow over time (i.e. the data on treatment-seeking), we did not find any change in patterns between year 2014 and 2021, indicating that the identified treatment gap remains.

### Implications

Effective treatments for eating disorders exist, but do not do much good if they do not reach those who need them. This study has identified a treatment gap in the Stockholm region, by which the substantially higher levels of eating disorder symptoms among people with a migration background is not matched by service utilisation. Strikingly, the five districts with the highest mean combined eating disorder scores – all of which also have a high or very high percentage of residents with a migration background – are all among the districts in which residents are least likely to receive treatment. This calls for oversight of the outreach strategies currently employed within the region and highlights the need for extension of the existing services, since available specialist eating disorder services have already been operating at maximum capacity for several years. Moreover, although this study was conducted in Stockholm County, there is nothing to suggest that the situation is any better in the rest of Sweden; arguably, the Stockholm region is privileged in terms of access to specialist eating disorder treatment compared with many other parts of the country.

Existing specialist eating disorder treatment frameworks may also need to be adapted so as to make them more relevant for groups from other cultural backgrounds than the majority population. Only a small number of studies have explored culturally tailored eating disorder treatment, and all of these describe how treatment programmes have been successfully adapted for the Latinx population in the USA through a greater focus on family involvement, the cultural importance of food as care, etc.^56,57^ A number of strategies for increasing treatment-seeking for groups with a migration background have been suggested,^58^ usually focused on community-based interventions tailored to individual migrant groups, and may be relevant for eating disorders. One straightforward way of highlighting culturally relevant aspects in treatment would be to integrate the use of the Cultural Formulation Interview (CFI), a person-centred instrument for the systematic exploration of cultural aspects in healthcare encounters, in specialist eating disorder assessment and treatment. To date, we know of only one study that makes use of the CFI in treatment of eating disorders: a case study of disordered eating in Ethiopian migrants in Israel.^59^ Importantly, the CFI can be used with all patients regardless of cultural background; not least, our relationship to food and eating is always saturated with sociocultural meaning, although it is sometimes approached as a strictly instrumental aspect of everyday life in eating disorder treatment programmes.

The identified treatment gap may also result in a knowledge gap. Most studies on disorders that tend to go unrecognised in the general population, such as eating disorders, need to be conducted in a clinical setting among existing patients. If study participants encountered in this setting are not representative for the entire group experiencing disordered eating, the findings will inevitably be less generalisable and relevant for the population at large. For example, it is uncertain if commonly used diagnostic instruments for eating disorder screening and assessment are valid and reliable in broader sections of society, since they have typically been tested mostly among those groups that do have access to specialist eating disorder treatment. Increasing access to adequate treatment for all individuals with an eating disorder is of utmost importance, both for their own well-being and for improving our knowledge of how disordered eating is experienced and expressed in all parts of society.

## Supporting information

Strand et al. supplementary material Strand et al. supplementary material

## Data Availability

Data are available from the corresponding author, M.S., upon reasonable request.
