# Supplementary material for: Migration background, eating disorder symptoms and healthcare service utilisation: findings from the Stockholm Public Health Cohort
Source: BJPsych Open. 2023 Nov 3;9(6):e205. doi: 10.1192/bjo.2023.599 (PMC10753962; doi:10.1192/bjo.2023.599)
Supplement: Strand et al. supplementary material [file S2056472423005999sup001.docx]

**Supplementary material**

| **Table S1. List of countries by geographic region** | | |  |  |  |  |
| --- | --- | --- | --- | --- | --- | --- |
| **Nordic countries (except Sweden)** | **Europe (except Nordic countries)** | **Africa** | **Asia** | **North America** | **South America** | **Oceania** |
| Denmark | Albania | Algeria | Afghanistan | Anguilla | Argentina | Australia |
| Finland | Andorra | Angola | Armenia | Antigua and Barbuda | Bolivia | Fiji |
| Iceland | Austria | Benin | Azerbajdzjan | Bahamas | Brazil | Kiribati |
| Norway | Belarus | Botswana | Bahrain | Barbados | Chile | Marshall Islands |
|  | Belgium | Burkina Faso | Bangladesh | Belize | Colombia | Micronesia |
|  | Bosnia and Herzegovina | Burundi | Bhutan | Bermuda | Ecuador | Nauru |
|  |  | Cameroon | Brunei | British Virgin Islands | Guyana | New Zeeland |
|  | Bulgaria | Central African Republic | Cambodia | Canada | Paraguay | Palau |
|  | Croatia |  | China | Costa Rica | Peru | Papua New Guinea |
|  | Cyprus | Chad | East Timor | Cuba | Suriname | Samoa |
|  | Czech Republic | Comoros | Georgia | Dominica | Uruguay | Solomon Islands |
|  | Estonia | Democratic Republic of the Congo | India | Dominican Republic | Venezuela | Tonga |
|  | France |  | Indonesia | El Salvador |  | Tuvalu |
|  | Germany | Djibouti | Iran | Grenada |  | Vanuatu |
|  | Gibraltar | Egypt | Iraq | Guatemala |  |  |
|  | Greece | Equatorial Guinea | Israel | Haiti |  |  |
|  | Holy See | Eritrea | Japan | Honduras |  |  |
|  | Hungary | Eswatini | Jordan | Jamaica |  |  |
|  | Ireland | Ethiopia | Kazakhstan | Mexico |  |  |
|  | Italy | Gabon | Kirgizistan | Nicaragua |  |  |
|  | Kosovo | Gambia | Kuwait | Panama |  |  |
|  | Latvia | Ghana | Laos | Saint Kitts and Nevis |  |  |
|  | Liechtenstein | Guinea | Libanon | Saint Lucia |  |  |
|  | Lithuania | Guinea-Bissau | Malaysia | Saint Vincent and the Grenadines |  |  |
|  | Luxembourg | Ivory Coast | Maldives |  |  |  |
|  | Malta | Kap Verde | Mongolia | Trinidad and Tobago |  |  |
|  | Moldavia | Kenya | Myanmar | United States |  |  |
|  | Monaco | Lesotho | Nepal |  |  |  |
|  | Montenegro | Liberia | North Korea |  |  |  |
|  | Netherlands | Libya | Oman |  |  |  |
|  | North Macedonia | Madagascar | Pakistan |  |  |  |
|  | Poland | Malawi | Palestina |  |  |  |
|  | Portugal | Mali | Philippines |  |  |  |
|  | Romania | Mauretania | Qatar |  |  |  |
|  | Russia | Mauritius | Saudi Arabia |  |  |  |
|  | San Marino | Moçambique | Singapore |  |  |  |
|  | Serbia | Morocco | South Korea |  |  |  |
|  | Slovakia | Namibia | Sri Lanka |  |  |  |
|  | Slovenia | Niger | Syria |  |  |  |
|  | Spain | Nigeria | Taiwan |  |  |  |
|  | Switzerland | Republic of the Congo | Tajikistan |  |  |  |
|  | Turkey | Rwanda | Thailand |  |  |  |
|  | Ukraine | São Tomé and | Turkmenistan |  |  |  |
|  | United Kingdom | Príncipe | United Arab |  |  |  |
|  |  | Senegal | Emirates |  |  |  |
|  |  | Seychelles | Uzbekistan |  |  |  |
|  |  | Sierra Leone | Vietnam |  |  |  |
|  |  | Somalia | Yemen |  |  |  |
|  |  | South Africa |  |  |  |  |
|  |  | South Sudan |  |  |  |  |
|  |  |  |  |  |  |  |
|  |  | Sudan |  |  |  |  |
|  |  | Tanzania |  |  |  |  |
|  |  | Togo |  |  |  |  |
|  |  | Tunisia |  |  |  |  |
|  |  | Uganda |  |  |  |  |
|  |  | Zambia |  |  |  |  |
|  |  | Zimbabwe |  |  |  |  |
|  |  |  |  |  |  |  |

| **Table S2. Description of the survey data by levels of exposure** | | | | |  |  |  |  |  |
| --- | --- | --- | --- | --- | --- | --- | --- | --- | --- |
|  | **Region of birth** | | | | | | | | |
|  | **Sweden** | | | **Europe (other than Sweden)** | | | **Non-European countries** | | |
|  | *n* | % | Weighted % | *n* | % | Weighted % | *n* | % | Weighted % |
| **Sex** |  |  |  |  |  |  |  |  |  |
| Female | 23128 | 56.6 | 49.4 | 2858 | 61.3 | 57.7 | 1122 | 53.5 | 51.7 |
| Male | 17727 | 43.4 | 50.6 | 1806 | 38.7 | 42.3 | 976 | 46.5 | 48.3 |
|  |  |  |  |  |  |  |  |  |  |
| **Age groups** |  |  |  |  |  |  |  |  |  |
| 22-29 years | 1355 | 3.3 | 19.2 | 39 | 0.8 | 3.8 | 60 | 2.9 | 7.6 |
| 30-44 years | 7544 | 18.5 | 32.5 | 534 | 11.4 | 18.3 | 537 | 25.6 | 29.9 |
| 45-66 years | 17587 | 43.0 | 30.7 | 2101 | 45.0 | 43.7 | 1227 | 58.5 | 52.0 |
| 67+ years | 14369 | 35.2 | 17.6 | 1990 | 42.7 | 34.1 | 274 | 13.1 | 10.5 |
|  |  |  |  |  |  |  |  |  |  |
|  | **Parent background** | | | | | | | | |
|  | **Both parents born in Sweden** | | | **One parent born abroad** | | | **Both parents born abroad** | | |
|  | *n* | % | Weighted % | *n* | % | Weighted % | *n* | % | Weighted % |
| **Sex** |  |  |  |  |  |  |  |  |  |
| Female | 17612 | 56.5 | 48.4 | 2705 | 57.3 | 51.0 | 4186 | 59.3 | 53.6 |
| Male | 13549 | 43.5 | 51.6 | 2014 | 42.7 | 49.0 | 2875 | 40.7 | 46.4 |
|  |  |  |  |  |  |  |  |  |  |
| **Age groups** |  |  |  |  |  |  |  |  |  |
| 22-29 years | 1041 | 3.3 | 18.7 | 214 | 4.5 | 24.6 | 201 | 2.8 | 10.0 |
| 30-44 years | 6175 | 19.8 | 34.4 | 952 | 20.2 | 32.3 | 1496 | 21.2 | 28.7 |
| 45-66 years | 14643 | 47.0 | 33.1 | 3201 | 48.8 | 32.3 | 3992 | 56.5 | 50.1 |
| 67+ years | 9302 | 29.9 | 13.7 | 1252 | 26.5 | 10.9 | 1372 | 19.4 | 11.2 |
|  |  |  |  |  |  |  |  |  |  |
|  |  |  |  |  |  |  |  |  |  |
|  |  |  |  |  |  |  |  |  |  |
|  | **Neighborhood population** | | | | | | | | |
|  | **<20% with migration background** | | | **20-40% with migration background** | | | **>40% with migration background** | | |
|  | *n* | % | Weighted % | *n* | % | Weighted % | *n* | % | Weighted % |
| **Sex** |  |  |  |  |  |  |  |  |  |
| Female | 10929 | 57.0 | 51.5 | 13907 | 57.0 | 50.5 | 2298 | 56.0 | 50.1 |
| Male | 8325 | 43.0 | 48.5 | 10485 | 43.0 | 49.5 | 1808 | 44.0 | 49.9 |
|  |  |  |  |  |  |  |  |  |  |
| **Age groups** |  |  |  |  |  |  |  |  |  |
| 22-29 years | 481 | 2.5 | 13.9 | 850 | 3.5 | 17.3 | 125 | 3.0 | 13.0 |
| 30-44 years | 3198 | 16.7 | 31.0 | 4769 | 19.6 | 31.0 | 656 | 16.0 | 25.5 |
| 45-66 years | 8484 | 44.2 | 34.8 | 10625 | 43.6 | 34.2 | 1827 | 44.5 | 41.2 |
| 67+ years | 7001 | 36.5 | 20.2 | 8148 | 33.4 | 17.5 | 1498 | 36.5 | 20.3 |
|  |  |  |  |  |  |  |  |  |  |

| **Table S3. General linear model including region of birth, sex, and age** | |
| --- | --- |
|  | B (95% CI) |
| **SCOFF3 score** |  |
| Europe (other than Sweden)^ⴕ^ | 0.08 (0.06, 0.10) |
| Non-European countries^ⴕ^ | 0.23 (0.19, 0.27) |
| Sex (female) | 0.10 (0.09, 0.12) |
| Age | -0.004 (-0.003, -0.004) |
| **Combined ED score** |  |
| Europe (other than Sweden)^ⴕ^ | 0.09 (0.07, 0.11) |
| Non-European countries^ⴕ^ | 0.32 (0.27, 0.36) |
| Sex (female) | 0.11 (0.09, 0.13) |
| Age | -0.003 (-0.004, -0.003) |
| **GHQ-12 score** |  |
| Europe (other than Sweden)^ⴕ^ | 0.13 (0.00, 0.25) |
| Non-European countries^ⴕ^ | 0.33 (0.17, 0.39) |
| Sex (female) | 0.50 (0.40, 0.59) |
| Age | -0.029 (-0.033, -0.026) |
| **Suicidality score** |  |
| Europe (other than Sweden)^ⴕ^ | 0.02 (0.00, 0.03) |
| Non-European countries^ⴕ^ | 0.01 (-0.01, 0.03) |
| Sex (female) | 0.04 (0.03, 0.05) |
| Age | -0.003 (-0.004, -0.003) |
| **BMI** |  |
| Europe (other than Sweden)^ⴕ^ | 0.60 (0.44, 0.77) |
| Non-European countries^ⴕ^ | 0.69 (0.45, 0.93) |
| Sex (female) | -1.29 (-1.42, -1.16) |
| Age | 0.038 (0.033, 0.042) |
|  |  |
| (BMI: Body Mass Index; CI: Confidence interval; ED: Eating disorder; GHQ-12: 12-Item General Health Questionnaire) | |
| ^ⴕ^Compared to the Swedish-born group as reference | |
|  |  |

| **Table S4. General linear model including parent background, sex, and age** | |
| --- | --- |
|  | B (95% CI) |
| **SCOFF3 score** |  |
| One parent born abroad^ⴕ^ | 0.03 (0.00, 0.06) |
| Both parents born abroad^ⴕ^ | 0.17 (0.14, 0.19) |
| Sex (female) | 0.11 (0.09, 0.13) |
| Age | -0.004 (-0.005, -0.003) |
| **Combined ED score** |  |
| One parent born abroad^ⴕ^ | 0.03 (0.00, 0.06) |
| Both parents born abroad^ⴕ^ | 0.22 (0.19, 0.24) |
| Sex (female) | 0.11 (0.09, 0.13) |
| Age | -0.004 (-0.004, -0.003) |
| **GHQ-12 score** |  |
| One parent born abroad^ⴕ^ | 0.19 (0.02, 0.37) |
| Both parents born abroad^ⴕ^ | 0.22 (0.11, 0.33) |
| Sex (female) | 0.49 (0.39, 0.59) |
| Age | -0.036 (-0.040, -0.032) |
| **Suicidality score** |  |
| One parent born abroad^ⴕ^ | 0.02 (0.00, 0.04) |
| Both parents born abroad^ⴕ^ | 0.02 (0.00, 0.03) |
| Sex (female) | 0.04 (0.03, 0.05) |
| Age | -0.003 (-0.004, -0.003) |
| **BMI** |  |
| One parent born abroad^ⴕ^ | 0.10 (-0.13, 0.32) |
| Both parents born abroad^ⴕ^ | 0.73 (0.54, 0.92) |
| Sex (female) | -1.33 (-1.46, -1.19) |
| Age | 0.053 (0.048, 0.058) |
|  |  |
| (BMI: Body Mass Index; CI: Confidence interval; ED: Eating disorder; GHQ-12: 12-Item General Health Questionnaire) | |
| ^ⴕ^Compared to the group with two parents born in Sweden as reference | |
|  |  |

| **Table S5. General linear model including neighborhood population, sex, and age** | |
| --- | --- |
|  | B (95% CI) |
| **SCOFF3 score** |  |
| 20-40% with migration background^ⴕ^ | 0.03 (0.02, 0.05) |
| >40% with migration background^ⴕ^ | 0.14 (0.11, 0.18) |
| Sex (female) | 0.11 (0.09, 0.13) |
| Age | -0.003 (-0.004, -0.003) |
| **Combined ED score** |  |
| 20-40% with migration background^ⴕ^ | 0.04 (0.03, 0.06) |
| >40% with migration background^ⴕ^ | 0.18 (0.14, 0.22) |
| Sex (female) | 0.11 (0.10, 0.13) |
| Age | -0.003 (-0.003, -0.002) |
| **GHQ-12 score** |  |
| 20-40% with migration background^ⴕ^ | 0.06 (-0.04, 0.15) |
| >40% with migration background^ⴕ^ | 0.20 (0.04, 0.36) |
| Sex (female) | 0.50 (0.41, 0.59) |
| Age | -0.029 (-0.032, -0.026) |
| **Suicidality score** |  |
| 20-40% with migration background^ⴕ^ | 0.00 (-0.01, 0.01) |
| >40% with migration background^ⴕ^ | 0.01 (-0.01, 0.03) |
| Sex (female) | 0.04 (0.03, 0.05) |
| Age | -0.003 (-0.004, -0.003) |
| **BMI** |  |
| 20-40% with migration background^ⴕ^ | 0.25 (0.12, 0.37) |
| >40% with migration background^ⴕ^ | 1.19 (0.95, 1.93) |
| Sex (female) | -1.26 (-1.39, -1.13) |
| Age | 0.040, 0.035, 0.044) |
|  |  |
| (BMI: Body Mass Index; CI: Confidence interval; ED: Eating disorder; GHQ-12: 12-Item General Health Questionnaire) | |
| ^ⴕ^Compared to the group with <20% population with migration background as reference | |
|  |  |

| **Table S6. Disordered eating, mental distress, body mass index, SCOFF items, and restricitive eating in individuals whose both parents are born in a non-European country** | | | |
| --- | --- | --- | --- |
|  | **Both parents born in Sweden** | **Both parents born in a non-European country** | |
|  | Mean | Mean | Mean difference^ⴕ^ (95% CI) |
| **Total population** |  |  |  |
| SCOFF3 score | 0.14 | 0.44 | 0.30 (0.17, 0.43) |
| Combined ED score | 0.16 | 0.53 | 0.38 (0.22, 0.53) |
| GHQ-12 score | 1.64 | 2.47 | 0.83 (0.38, 1.27) |
| Suicidality score | 0.15 | 0.22 | 0.08 (0.01, 0.14) |
| BMI | 25.0 | 25.4 | 0.48 (-0.76, 1.72) |
|  |  |  |  |
| **Females** |  |  |  |
| SCOFF3 score | 0.19 | 0.57 | 0.38 (0.18, 0.58) |
| Combined ED score | 0.22 | 0.67 | 0.46 (0.21, 0.70) |
| GHQ-12 score | 1.84 | 3.05 | 1.21 (0.56, 1.86) |
| Suicidality score | 0.16 | 0.29 | 0.13 (0.03, 0.22) |
| BMI | 24.3 | 24.2 | -0.18 (-0.82, 0.42) |
|  |  |  |  |
| **Males** |  |  |  |
| SCOFF3 score | 0.09 | 0.28 | 0.19 (0.06, 0.33) |
| Combined ED score | 0.10 | 0.37 | 0.27 (0.12, 0.42) |
| GHQ-12 score | 1.46 | 1.74 | 0.29 (-0.24, 0.82) |
| Suicidality score | 0.14 | 0.15 | 0.01 (-0.08, 0.10) |
| BMI | 25.6 | 26.9 | 1.38 (-1.09, 3.85) |
|  |  |  |  |
|  | % "Yes" | % "Yes" | OR^ⴕ^ (95% CI) |
| **SCOFF item** |  |  |  |
| Compensatory vomiting | 0.9 | 4.9 | 5.8 (2.6, 12.9) |
| Loss-of-control eating | 7.6 | 25.4 | 4.2 (2.9, 6.0) |
| Preoccupation with food | 5.4 | 13.3 | 2.7 (1.7, 4.4) |
| **Restrictive eating** | 2.0 | 9.0 | 4.8 (2.5, 9.0) |
|  |  |  |  |
| (BMI: Body Mass Index; CI: Confidence interval; ED: Eating disorder; GHQ-12: 12-Item General Health Questionnaire; OR: Odds ratio) | | | |
| ^ⴕ^Compared to the group with two parents born in Sweden as reference | | | |
|  |  |  |  |

| **Table S7. Disordered eating, mental distress, body mass index, SCOFF items, and restricitive eating according to birth region** | | | | | |
| --- | --- | --- | --- | --- | --- |
|  | **Sweden** | **Nordic countries (except Sweden)** | | **Europe (except Nordic countries)** | |
|  | Mean | Mean | Mean difference^ⴕ^ (95% CI) | Mean | Mean difference^ⴕ^ (95% CI) |
| SCOFF3 score | 0.15 | 0.16 | 0.01 (-0.02, 0.04) | 0.24 | 0.09 (0.06, 0.12) |
| Combined ED score | 0.17 | 0.18 | 0.02 (-0.02, 0.05) | 0.28 | 0.11 (0.08, 0.15) |
| GHQ-12 score | 1.67 | 1.30 | -0.38 (-0.24, -0.21) | 1.75 | 0.08 (-0.11, 0.26) |
| Suicidality score | 0.15 | 0.12 | -0.03 (-0.05, -0.01) | 0.15 | 0.00 (-0.03, 0.02) |
| BMI | 25.0 | 26.0 | 1.04 (0.82, 1.27) | 25.7 | 0.77 (0.53, 1.01) |
|  |  |  |  |  |  |
|  | % "Yes" | % "Yes" | OR^ⴕ^ (95% CI) | % "Yes" | OR^ⴕ^ (95% CI) |
| **SCOFF item** |  |  |  |  |  |
| Compensatory vomiting | 1.1 | 2.0 | 1.9 (1.0, 3.5) | 3.2 | 3.1 (2.2, 4.4) |
| Loss-of-control eating | 7.9 | 7.6 | 1.0 (0.8, 1.2) | 11.7 | 1.6 (1.3, 1.9) |
| Preoccupation with food | 5.7 | 6.2 | 1.1 (0.9, 1.4) | 8.7 | 1.6 (1.3, 2.0) |
| **Restrictive eating** | 2.2 | 2.9 | 1.3 (1.0, 1.8) | 5.1 | 2.3 (1.8, 3.1) |
|  |  |  |  |  |  |
|  |  |  |  |  |  |
|  |  |  |  |  |  |
|  |  |  |  |  |  |
|  |  |  |  |  |  |
|  |  |  |  |  |  |
|  |  |  |  |  |  |
|  |  |  |  |  |  |
|  |  |  |  |  |  |
|  |  |  |  |  |  |
|  |  |  |  |  |  |
|  |  |  |  |  |  |
|  |  |  |  |  |  |
|  |  |  |  |  |  |
|  |  | **Africa** | | **Asia** | |
|  |  | Mean | Mean difference^ⴕ^ (95% CI) | Mean | Mean difference^ⴕ^ (95% CI) |
| SCOFF3 score |  | 0.27 | 0.13 (0.05, 0.21) | 0.44 | 0.30 (0.24, 0.35) |
| Combined ED score |  | 0.42 | 0.25 (0.16, 0.34) | 0.56 | 0.39 (0.33, 0.46) |
| GHQ-12 score |  | 1.89 | 0.22 (-0.17, 0.62) | 1.99 | 0.32 (0.11, 0.53) |
| Suicidality score |  | 0.11 | -0.04 (-0.08, 0.00) | 0.15 | 0.01 (-0.02, 0.03) |
| BMI |  | 25.9 | 0.96 (0.46, 1.46) | 25.6 | 0.66 (0.32, 1.00) |
|  |  |  |  |  |  |
|  |  | % "Yes" | OR^ⴕ^ (95% CI) | % "Yes" | OR^ⴕ^ (95% CI) |
| **SCOFF item** |  |  |  |  |  |
| Compensatory vomiting |  | 6.0 | 6.0 (3.4, 10.6) | 7.5 | 7.6 (5.5, 10.3) |
| Loss-of-control eating |  | 12.0 | 1.6 (1.1, 2.4) | 21.7 | 3.2 (2.7, 3.9) |
| Preoccupation with food |  | 10.1 | 1.9 (1.2, 2.9) | 15.0 | 2.9 (2.4, 3.6) |
| **Restrictive eating** |  | 14.7 | 7.5 (5.2, 10.9) | 11.3 | 5.6 (4.3, 7.2) |
|  |  |  |  |  |  |
|  |  |  |  |  |  |
|  |  |  |  |  |  |
|  |  | **North America** | | **South America** | |
|  |  | Mean | Mean difference^ⴕ^ (95% CI) | Mean | Mean difference^ⴕ^ (95% CI) |
| SCOFF3 score |  | 0.24 | 0.10 (-0.02, 0.21) | 0.25 | 0.10 (0.04, 0.17) |
| Combined ED score |  | 0.32 | 0.16 (0.03, 0.28) | 0.34 | 0.17 (0.10, 0.25) |
| GHQ-12 score |  | 1.60 | -0.08 (-0.57, 0-42) | 2.10 | 0.43 (0.03, 0.83) |
| Suicidality score |  | 0.16 | 0.01 (-0.06, 0.07) | 0.18 | 0.03 (-0.02, 0.08) |
| BMI |  | 25.6 | 0.66 (-0.07, 1.39) | 25.9 | 0.97 (0.48, 1.46) |
|  |  |  |  |  |  |
|  |  | % "Yes" | OR^ⴕ^ (95% CI) | % "Yes" | OR^ⴕ^ (95% CI) |
| **SCOFF item** |  |  |  |  |  |
| Compensatory vomiting |  | 0.8 | 0.7 (0.2, 3.1) | 3.9 | 3.8 (2.0, 7.0) |
| Loss-of-control eating |  | 14.2 | 1.9 (1.1, 3.3) | 14.4 | 2.0 (1.4, 2.8) |
| Preoccupation with food |  | 9.0 | 1.6 (0.8, 3.1) | 6.2 | 1.1 (0.6, 1.8) |
| **Restrictive eating** |  | 8.7 | 4.2 (2.2, 7.9) | 9.0 | 4.3 (2.9, 6.4) |
|  |  |  |  |  |  |
|  |  | **Oceania** | |  |  |
|  |  | Mean | Mean difference^ⴕ^ (95% CI) |  |  |
| SCOFF3 score |  | 0.04 | -0.10 (-0.19, -0.01) |  |  |
| Combined ED score |  | 0.04 | -0.12 (-0.21, -0.04) |  |  |
| GHQ-12 score |  | 0.53 | -1.15 (-1.60, -0.69) |  |  |
| Suicidality score |  | 0.00 | -0.15 (-0.16, -0.14) |  |  |
| BMI |  | 26.3 | 1.32 (-2.01, 4.65) |  |  |
|  |  |  |  |  |  |
|  |  | % "Yes" | OR^ⴕ^ (95% CI) |  |  |
| **SCOFF item** |  |  |  |  |  |
| Compensatory vomiting |  | 0.0 | n/a |  |  |
| Loss-of-control eating |  | 4.5 | 0.5 (0.1, 4.2) |  |  |
| Preoccupation with food |  | 0.0 | n/a |  |  |
| **Restrictive eating** |  | 0.0 | n/a |  |  |
|  |  |  |  |  |  |
| (BMI: Body Mass Index; CI: Confidence interval; ED: Eating disorder; GHQ-12: 12-Item General Health Questionnaire; OR: Odds ratio) | | | | | |
| ^ⴕ^Compared to Swedish-born group as reference | |  |  |  |  |


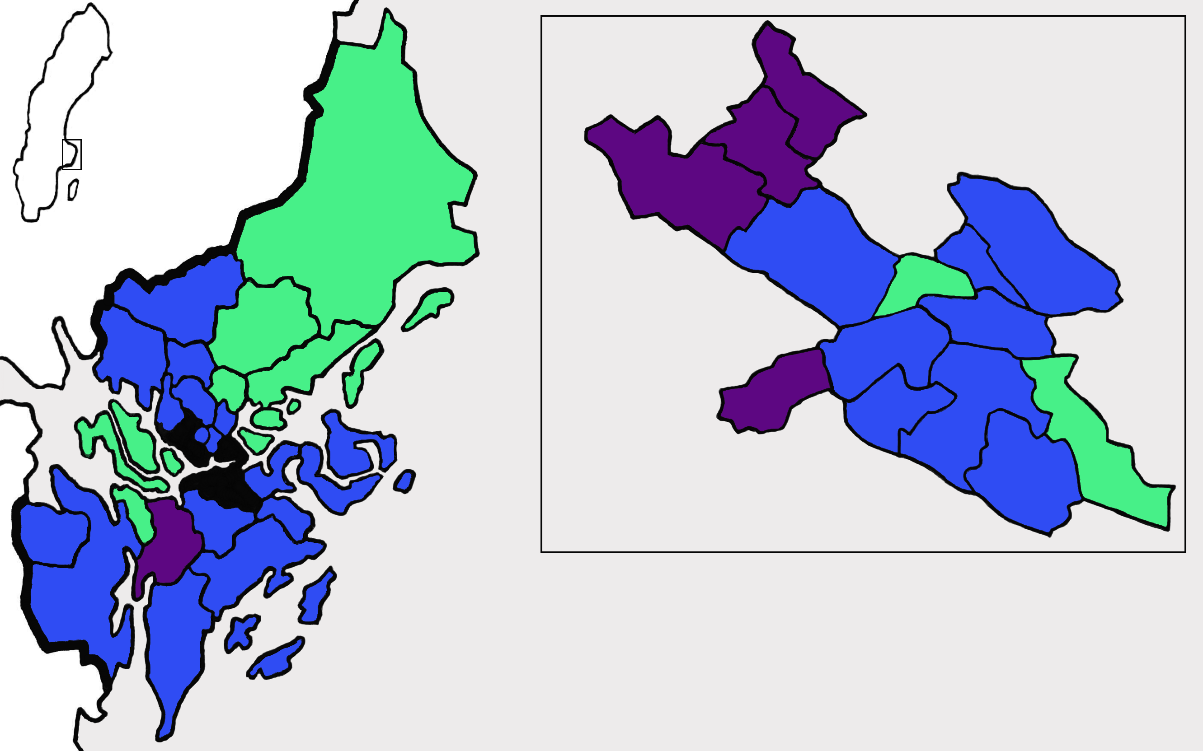


A. Eating disorder symptoms


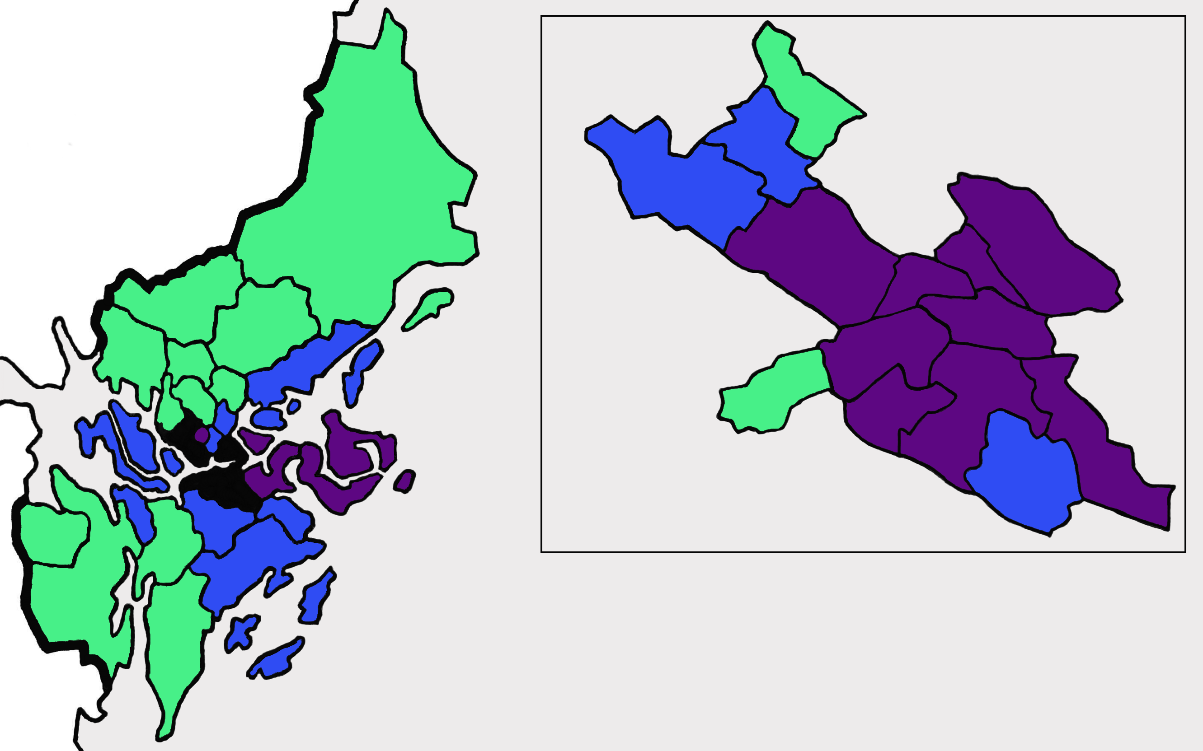


B. Treatment seeking


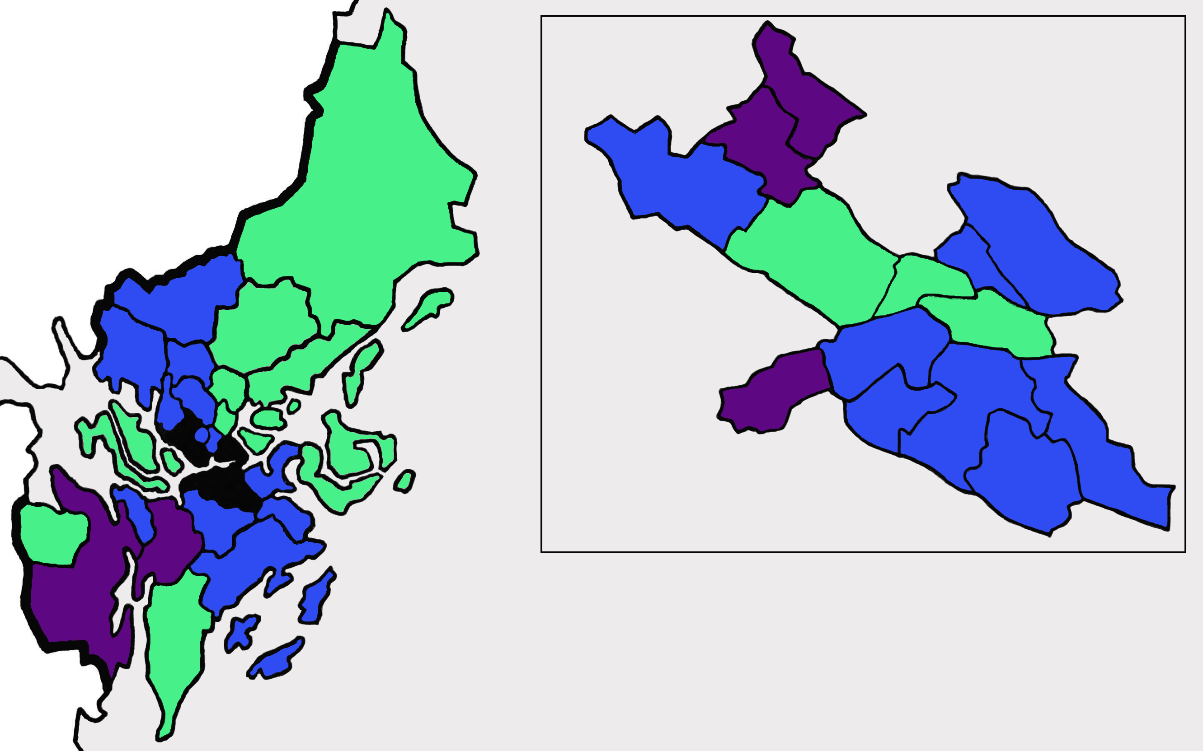


C. Population with migration background

Around county average

Clearly below county average

Clearly above county average

Supplemental figure 1. Eating disorder symptoms (A), treatment seeking patterns (B), and proportion of population with a migration background in Stockholm county (with the City of Stockholm shown as inset). The Stockholm County is divided into 26 municipalities. One of these municipalities, the City of Stockholm—the most populous municipality in Sweden—is further divided into 14 districts. Thus, a total of 39 geographic strata were used in this study.
